# Intoxicación escombroide secundaria al consumo de atún: presentación de un caso

**DOI:** 10.7705/biomedica.5283

**Published:** 2020-12-09

**Authors:** María Carolina González, Andrea Carolina Díaz, Jairo Giovanni Moncayo, Jorge Alonso Marín

**Affiliations:** 1 Servicio de Urgencias, Hospital Mental de Antioquia, Bello, Colombia Hospital Mental de Antioquia Bello Colombia; 2 Servicio de Urgencias, Clínica SOMA, Medellín, Colombia Clínica SOMA Medellín Colombia; 3 Departamento de Medicina de Urgencias, Universidad CES, Medellín, Colombia Universidad CES Departamento de Medicina de Urgencias Universidad CES Medellín Colombia; 4 Servicio de Urgencias, Clínica CES, Medellín, Colombia Clínica CES Medellín Colombia; 5 Servicio de Toxicología Clínica, Clínica SOMA, Medellín, Colombia Clínica SOMA Medellín Colombia; 6 Servicio de Toxicología Clínica, Hospital Marco Fidel Suárez, Bello, Colombia Hospital Marco Fidel Suárez Bello Colombia; 7 Facultad de Medicina, Corporación Universitaria Remington, Medellín, Colombia Corporación Universitaria Remington Facultad de Medicina Corporación Universitaria Remington Medellín Colombia; 8 Grupo Infettare, Facultad de Medicina, Universidad Cooperativa de Colombia, Medellín, Colombia Universidad Cooperativa de Colombia Facultad de Medicina Universidad Cooperativa de Colombia Medellín Colombia

**Keywords:** enfermedades transmitidas por los alimentos, atún, histamina, arritmias cardíacas, Foodborne diseases, tuna, histamine, arrhythmias, cardiac

## Abstract

La intoxicación escombroide es ocasionada por el consumo de ciertos tipos de pescado (de la familia *Scombridae),* comúnmente el atún, los cuales acumulan grandes concentraciones de histamina cuando los procedimientos de refrigeración son inadecuados, ocasionando en quienes los consumen síntomas muy similares a los de una alergia alimentaria, por lo que es frecuente que no se diagnostique correctamente. Generalmente, los síntomas desaparecen en pocas horas y no suelen ser graves, excepto algunos casos descritos en la literatura especializada, de hipotensión, broncoespasmo, dificultad respiratoria, taquicardia supraventricular e, incluso, infarto agudo de miocardio.

Este fue, precisamente, el caso de una mujer que ingresó al servicio de urgencias de un hospital de tercer nivel de Medellín a los pocos minutos de haber ingerido atún con una sintomatología típica de la intoxicación, pero con taquicardia supraventricular, una de sus manifestaciones graves y atípicas.

## Abstract (en)

Scombroid poisoning is caused by the consumption of certain types of fish (from the *Scombridae* family), especially tuna. Due to inadequate refrigeration procedures, these fish have high levels of histamine which generate symptoms similar to those of a food allergy in their consumers, so it is frequently underdiagnosed. It is self-limited in a few hours and the symptoms are usually not serious, except for specific cases reported in the literature of hypotension, bronchospasm, respiratory distress, tachyarrhythmias, and even acute myocardial infarction.

We report here the case of a woman admitted to the emergency department of a third level hospital in Medellín a few minutes after eating tuna with the typical symptoms of intoxication, as well as tachyarrhythmias, a serious and atypical manifestation.

La intoxicación escombroide es ocasionada por el consumo de ciertos tipos de pescado refrigerados de forma inadecuada ^1^. Los síntomas son similares a los de las alergias a la comida de mar, por lo que frecuentemente el diagnóstico es equivocado ^2^^,^^3^. Su nombre se debe a que se presenta en mayor medida con el consumo de pescados de la familia *Scombridae* (atún, bonito, caballa, entre otros) ^2^^,^^4^, que tienen mayores concentraciones de histidina, la cual se convierte en histamina por la acción de la enzima histidina descarboxilasa presente en las bacterias que residen en las branquias y el tubo digestivo de este tipo de peces y cuyo crecimiento es mayor a temperaturas entre los 20 y los 30 °C ^5^, por lo que el pescado debe conservarse a 0 °C o menos para evitar su proliferación.

Entre el 2009 y el 2012, se reportaron 40 brotes de la enfermedad en Estados Unidos, los cuales afectaron a 136 personas. También se reporta frecuentemente en Gran Bretaña y Japón; en este último país, ocurrió el brote con mayor número de afectados, 2.656, en 1973.

Los síntomas aparecen generalmente a los 20 o 30 minutos del consumo e incluyen rubor facial, dolor abdominal, diarrea, cefalea, palpitaciones, náuseas, vómito, boca seca, malestar general y mareo, los cuales desaparecen entre seis y ocho horas después ^6^^,^^7^. Se pueden presentar reacciones graves, como hipotensión arterial, broncoespasmo, dificultad respiratoria, arritmias (fibrilación auricular, taquicardia supraventricular y bloqueo auriculoventricular ^8^ e, incluso, infarto agudo de miocardio ^6^^,^^9^.

## Caso clínico

Se presenta el caso de una mujer de 33 años con hipertensión arterial crónica no tratada farmacológicamente y sin antecedentes alérgicos conocidos. Ingresó al servicio de urgencias de una clínica de tercer nivel de Medellín 30 minutos después de ingerir un filete de atún, con los siguientes síntomas: odinofagia, disnea, edema en labios, dolor abdominal, y calor y enrojecimiento facial.

En el examen físico, la paciente presentaba taquicardia con una frecuencia cardiaca de 160 latidos por minuto, la tensión arterial era normal y la saturación de oxígeno adecuada para el aire ambiente, estaba afebril y tenía rubicundez en cara y tórax anterior, pero sin evidencia de ruidos respiratorios anormales en la auscultación pulmonar.

**Figura 1:**
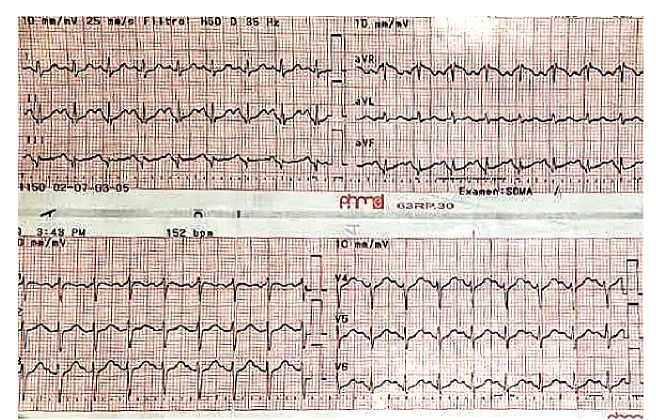
Taquicardia de complejos estrechos, regular, frecuencia cardíaca de 150 latidos por minuto, eje normal, sin alteraciones en repolarización ni signos de isquemia miocárdica

Se obtuvo un electrocardiograma que reveló una taquicardia supraventricular (figura 1), la cual se revirtió utilizando la maniobra de Valsalva: se sentó a la paciente y se le pidió que intentara sacar el émbolo de una jeringa de 10 ml soplando durante 15 segundos, luego se la acostó rápidamente y se elevaron sus piernas a 45° ^10^. La maniobra fue exitosa, y el ritmo sinusal y la frecuencia cardiaca se normalizaron en 90 latidos por minuto, verificados mediante monitoreo electrocardiográfico. Se inició la administración de esteroides orales, antihistamínicos e hidratación, y se solicitaron exámenes de laboratorio.

A las dos horas, consultaron en el mismo servicio de urgencias dos compañeras de trabajo de esta paciente que también habían consumido atún y presentaban una sintomatología similar, pero con manifestaciones menos graves, pues presentaban estabilidad hemodinámica y no había alteración del ritmo en el electrocardiograma. Los tres casos fueron reportados al sistema de vigilancia en salud pública de Medellín.

**Figura 2:**
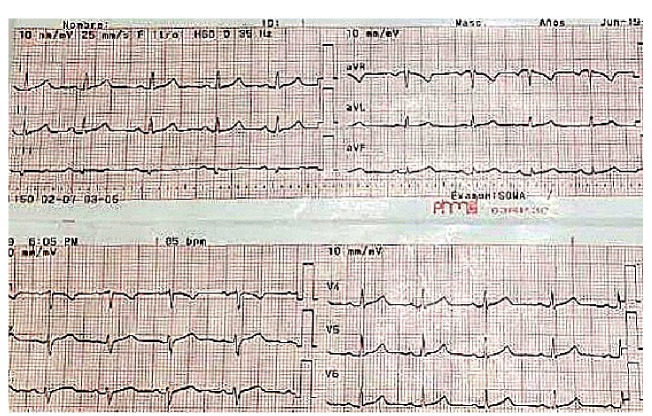
Ritmo sinusal, frecuencia cardíaca de 78 latidos por minuto, eje normal, electrocardiograma sin hallazgos patológicos

Después del tratamiento inicial, la paciente tuvo una adecuada evolución clínica; en los exámenes de laboratorio se evidenció la conservación de la función renal, no se registraron alteraciones de la función hepática, los gases arteriales indicaban una leve alcalosis respiratoria, no había alteración electrolítica y los rangos de creatina-fosfocinasa eran normales; en los electrocardiogramas de control cada 12 horas, persistía el ritmo sinusal (figuras 2 y 3), la paciente estaba asintomática y fue dada de alta al día siguiente.

**Figura 3:**
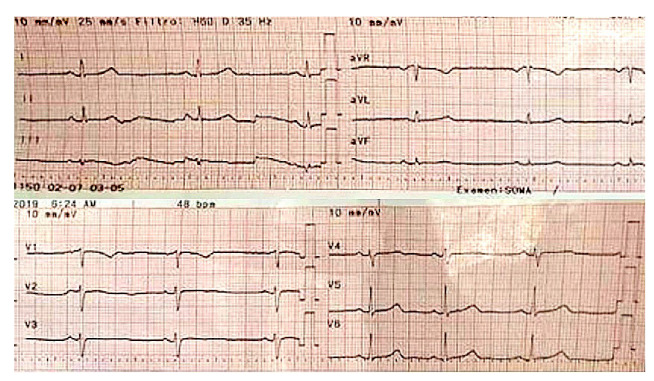
Bradicardia sinusal de 45 latidos por minuto, eje normal, sin signos de bloqueo aurículo-ventricular ni trastorno de repolarización

### Consideraciones éticas

Se obtuvo la aprobación y el consentimiento de la paciente para publicar su caso.

## Discusión

Se consideró que la paciente presentaba una intoxicación escombroide con base en el cuadro clínico ^11^, pues la sintomatologia correspondía a la que comúnmente se encuentra en estos casos. Además, había consumido el tipo de pescado que la produce y hubo otros dos casos de personas que también lo consumieron y tuvieron síntomas similares. Otros factores que favorecieron el diagnóstico fue la rapidez (menos de 30 minutos) con que aparecieron los síntomas después del consumo ^12^ y su rápida resolución con el tratamiento antihistamínico ^3^.

En general, la paciente presentó síntomas de una intoxicación leve, pero algunos poco comunes que solo se dan en intoxicaciones graves, como la taquicardia supraventricular. La etiología de las arritmias en este tipo de intoxicación puede explicarse por el efecto de la histamina en los receptores H_1_ y H_2_ de las fibras automáticas de las aurículas izquierda y derecha, respectivamente. La estimulación del automatismo ectópico y del nódulo sinusal depende de los receptores H_2_ y las alteraciones en la conducción son mediadas por los receptores H_1_ (enlentecimiento de la conducción auriculoventricular). Además, la histamina favorece la aparición de despolarizaciones tardías, las cuales, a su vez, propician la actividad desencadenada ^8^^,^^13^ y tienen un efecto cronotrópico positivo ^13^^-^^15^.

La histamina no está presente en los peces de forma natural, se produce después de su muerte cuando se inhiben sus mecanismos de defensa contra la proliferación bacteriana. La conversión de histidina en histamina se da en etapas tempranas de la descomposición cuando, aparentemente, el pescado todavía es comestible y no presenta cambios en su olor o coloración. La cocción del pescado a altas temperaturas solo evita la conversión de histidina a histamina, pero no elimina la histamina que se ha producido con anterioridad ^16^, por lo que el riesgo de intoxicación sigue latente y la persona puede presentar los mismos síntomas que provoca el pescado contaminado.

La *Food and Drug Administration* (FDA) de los Estados Unidos considera que concentraciones de histamina de 50 mg por 100 g de pescado son tóxicos. La mayoría de los individuos presenta cuadros clínicos de intoxicación cuando las concentraciones de histamina alcanzan los 100 mg/100 g de pescado; sin embargo, se han notificado casos graves con concentraciones de 20 mg/100 g de pescado en personas propensas ^16^.

El almacenamiento del pescado a temperaturas por encima de los 10 °C facilita la actividad de la enzima histidina carboxilasa producida por ciertas bacterias de los géneros *Proteus* sp., *Klebsiella* sp., *Aerobacter* sp. y por *Escherichia coli*^15^, y favorece la liberación de histamina hasta alcanzar rápidamente niveles tóxicos y producir efectos deletéreos en la salud de quienes consumen estos pescados.

La variabilidad del espectro clínico de los pacientes intoxicados se puede explicar por la propensión del individuo a la infección, el estado de su sistema inmunitario y la forma en que este responde a un alérgeno determinado. Pueden aparecer cuadros clínicos leves, con síntomas de intolerancia gastrointestinal y manifestaciones cutáneas, o graves y potencialmente fatales, con toxicidad cardiovascular como el choque anafiláctico, las arritmias y el síndrome coronario agudo debido al depósito de histamina en el miocardio ^15^. El tratamiento de la intoxicación se basa en el uso de antihistamínicos anti-H_1_ y anti-H_2_, esteroides intravenosos, líquidos endovenosos y epinefrina en caso de choque o manifestaciones graves, así como el tratamiento sintomático y específico de cada arritmia en caso de presentarse.

En conclusión, la intoxicación escombroide debe sospecharse en pacientes con manifestaciones sistémicas que se inicien después de ingerir pescado. Debe averiguarse cómo se almacenaron los alimentos antes de su consumo y si hubo cambios en su olor o sabor. El diagnóstico de esta condición es clínico dadas las limitaciones de tiempo y las dificultades para obtener muestras adecuadas para detectar los niveles de histamina, por lo que debe hacerse un interrogatorio cuidadoso para determinar la posible causa de la intoxicación y ofrecer un tratamiento temprano que evite resultados potencialmente mortales.
